# Systems biology analysis of osteogenic differentiation behavior by canine mesenchymal stem cells derived from bone marrow and dental pulp

**DOI:** 10.1038/s41598-020-77656-0

**Published:** 2020-11-26

**Authors:** Sirirat Nantavisai, Trairak Pisitkun, Thanaphum Osathanon, Prasit Pavasant, Chanin Kalpravidh, Sirakarnt Dhitavat, Jiradej Makjaroen, Chenphop Sawangmake

**Affiliations:** 1 Graduate Program in Veterinary Bioscience, Faculty of Veterinary Science, Chulalongkorn University, Bangkok, Thailand; 2 Veterinary Clinical Stem Cell and Bioengineering Research Unit, Faculty of Veterinary Science, Chulalongkorn University, Bangkok, Thailand; 3 Veterinary Stem Cell and Bioengineering Innovation Center (VSCBIC), Veterinary Pharmacology and Stem Cell Research Laboratory, Faculty of Veterinary Science, Chulalongkorn University, Bangkok, Thailand; 4 Center of Excellence in Systems Biology, Research Affairs, Faculty of Medicine, Chulalongkorn University, Bangkok, Thailand; 5 Department of Anatomy, Faculty of Dentistry, Chulalongkorn University, Bangkok, Thailand; 6 Center of Excellence for Regenerative Dentistry, Chulalongkorn University, Bangkok, Thailand; 7 Department of Surgery, Faculty of Veterinary Science, Chulalongkorn University, Bangkok, Thailand; 8 Biochemistry Unit, Department of Physiology, Faculty of Veterinary Science, Chulalongkorn University, Bangkok, Thailand; 9 Department of Pharmacology, Faculty of Veterinary Science, Chulalongkorn University, Bangkok, Thailand

**Keywords:** Stem cells, Systems biology

## Abstract

Utilization of canine mesenchymal stem cells (cMSCs) for regenerating incorrigible bone diseases has been introduced. However, cMSCs harvested from different sources showed distinct osteogenicity. To clarify this, comparative proteomics-based systems biology analysis was used to analyze osteogenic differentiation behavior by cMSCs harvested from bone marrow and dental pulp. The results illustrated that canine dental pulp stem cells (cDPSCs) contained superior osteogenicity comparing with canine bone marrow-derived MSCs (cBM-MSCs) regarding alkaline phosphatase activity, matrix mineralization, and osteogenic marker expression. Global analyses by proteomics platform showed distinct protein clustering and expression pattern upon an in vitro osteogenic induction between them. Database annotation using Reactome and DAVID revealed contrast and unique expression profile of osteogenesis-related proteins, particularly on signaling pathways, cellular components and processes, and cellular metabolisms. Functional assay and hierarchical clustering for tracking protein dynamic change confirmed that cBM-MSCs required the presences of Wnt, transforming growth factor (TGF)-beta, and bone-morphogenetic protein (BMP) signaling, while cDPSCs mainly relied on BMP signaling presentation during osteogenic differentiation in vitro. Therefore, these findings illustrated the comprehensive data regarding an in vitro osteogenic differentiation behavior by cBM-MSCs and cDPSCs which is crucial for further mechanism study and the establishment of cMSC-based bone tissue engineering (BTE) for veterinary practice.

## Introduction

Regenerative therapy for reconstructing craniofacial and bone defects is a challenge procedure especially for veterinary practice^1^. This is due to the limited understanding of mesenchymal stem cells (MSCs) used as osteogenic precursor^2^. Canine bone marrow-derived MSCs (cBM-MSCs) and canine dental pulp stem cells (cDPSCs) are among the potential candidate suitable for further application according to their availability and accessibility^3^. Several studies have reported their characteristics and differentiation potential, but none of the study clarifies the comparative properties focusing on osteogenic differentiation potential^2,4,5^. cBM-MSCs and cDPSCs are MSCs respectively derived from bone marrow aspirate and dental pulp tissue that naturally serve as osteogenic or odontogenic precursor of their tissue origins^6,7^. Mostly, the in vitro studies described their osteogenic differentiation potential based on alkaline phosphatase activity, extracellular matrix (ECM) mineralization, osteogenic marker expression, and optimization of induction protocol without further detail on governing signaling cascades and potential application analysis^8,9^. Recent studies using omics-based systems biology approach have thoroughly dissected the osteogenic differentiation behavior of BM-MSCs and DPSCs from human resources suggesting the correlated and uncorrelated characteristics upon an in vitro induction model^10,11^. These data efficiently demonstrated the potential osteogenic regulating pathways referring the potential application of cells in further in vivo models^12–14^. Thusly, this kind of information is crucial for progressive development of cMSC-based bone tissue engineering (BTE) in veterinary practice. In this study, comparative global analysis regarding the in vitro osteogenic differentiation behavior of cBM-MSCs and cDPSCs was performed using proteomics-based systems biology approach along with functional confirming assay and hierarchical clustering analysis of the potential osteogenic-regulating pathways.

## Results

### cBM-MSCs and cDPSCs revealed MSC-like properties

**Figure 1:**
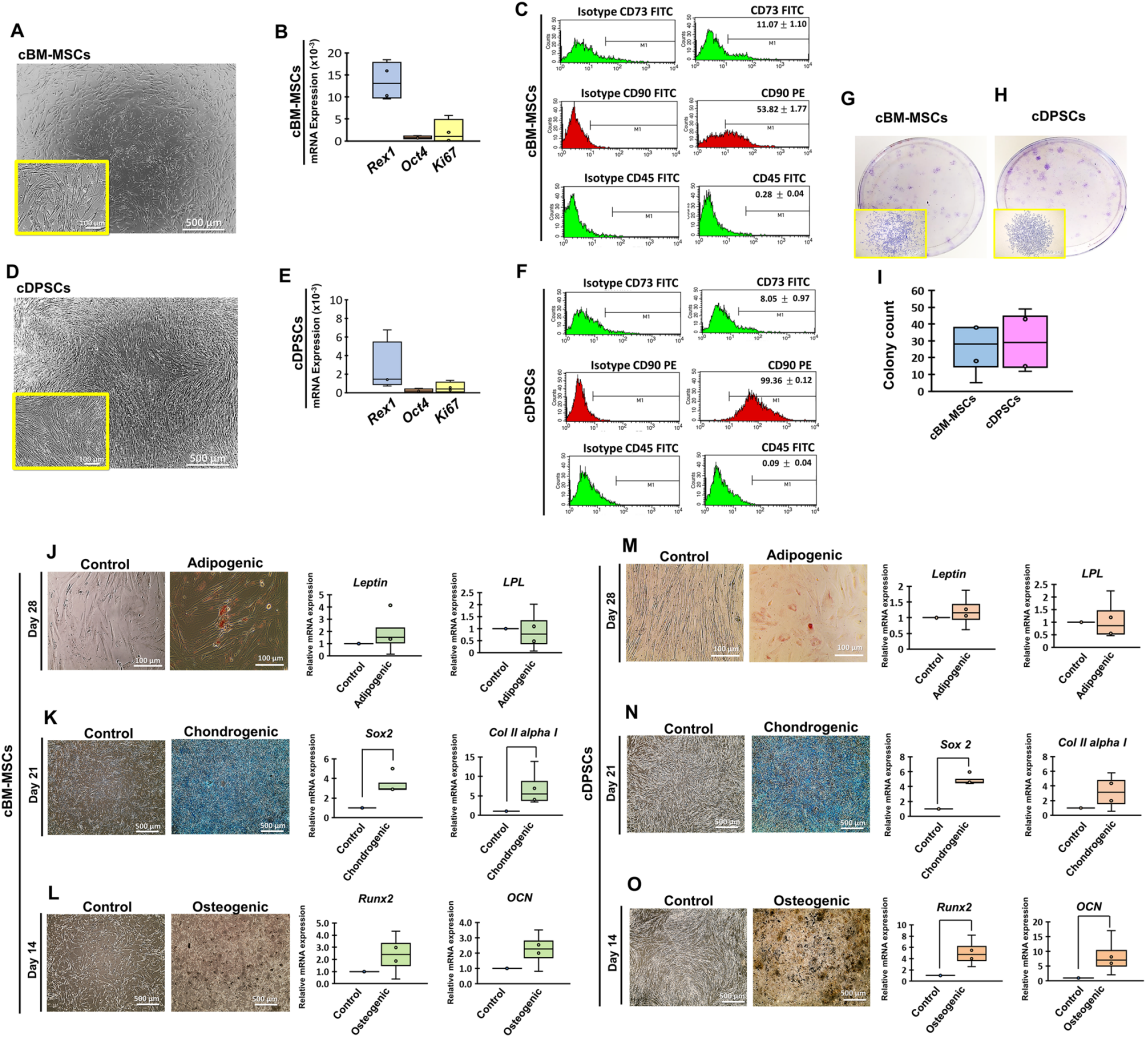
Morphology and characterization of cBM-MSCs and cDPSCs. Morphology (representative figures) (**A**,**D**), the expression of mRNA related stemness and proliferative markers (**B**,**E**), cell surface markers analysis (**C**,**F**), colony-forming capability and colony count (**G**–**I**), and multi-lineage differentiation potential regarding adipogenicity (**J**,**M**), chondrogenicity (**K**,**N**), and osteogenicity (**L**,**O**) of the isolated cBM-MSCs and cDPSCs were illustrated (n = 4). Relative mRNA expression of stemness and proliferative markers was normalized with the reference gene, *Gapdh*. The expression of mRNA markers relating multi-lineage induction was normalized with the reference gene and the undifferentiated control. Bars indicated the significant difference between groups (*p* value < 0.05).

The isolated cBM-MSCs and cDPSCs were attachment-dependent, fibroblast-like cells with the mRNA expression relating stemness (*Rex1* and *Oct4*) and proliferative (*Ki67*) properties (Fig. 1A,B,D,E). Flow cytometry analysis revealed the expression of MSC-related surface markers (CD73 and CD90) and the absence of hematopoietic cell marker (CD45) (Fig. 1C,F). Colony-forming capability (Fig. 1G–I) and the multi-lineage differentiation potential toward adipogenic, chondrogenic, and osteogenic lineages were illustrated (Fig. 1J–O).

### cBM-MSCs and cDPSCs possessed different osteogenic differentiation potential in vitro

**Figure 2:**
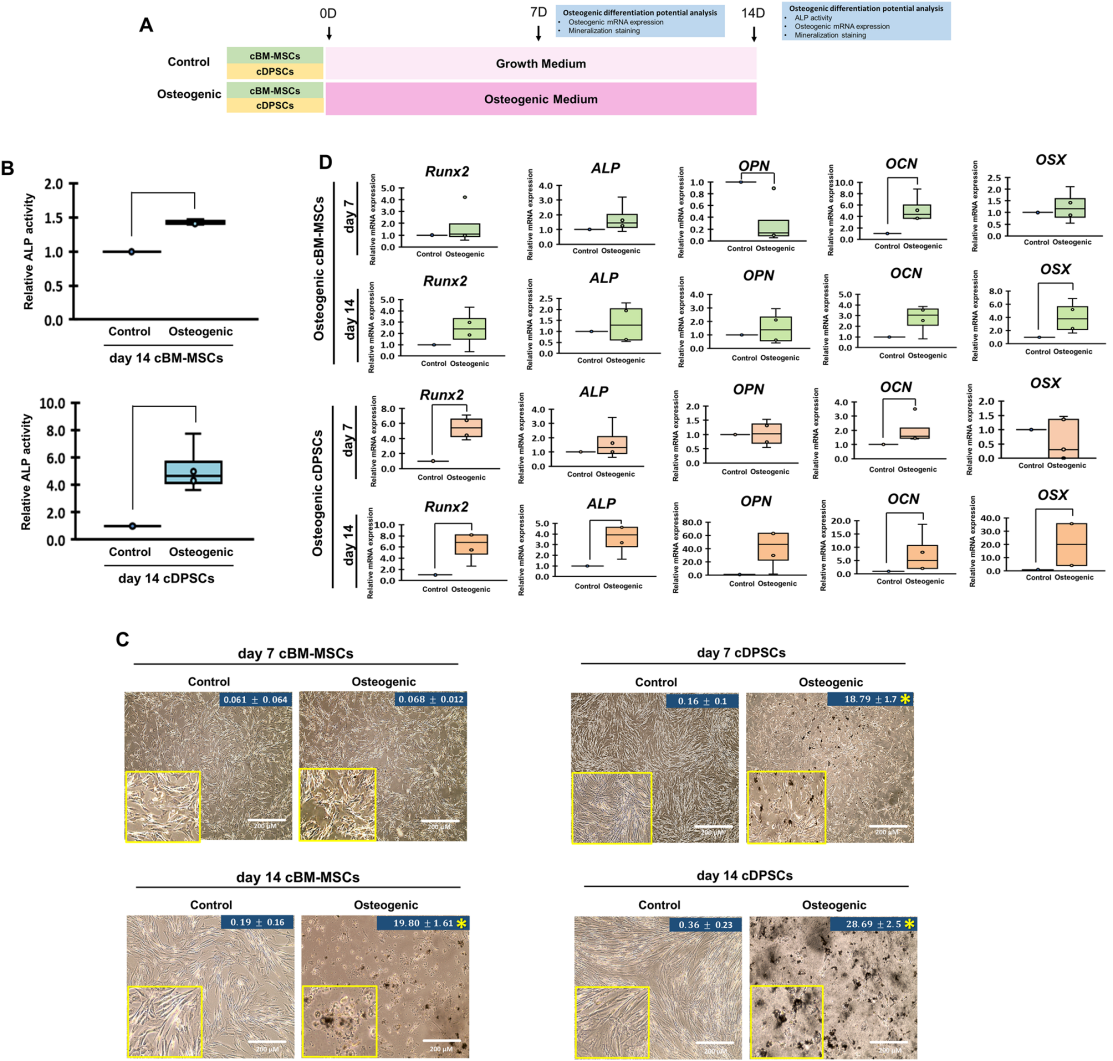
cDPSCs contained a superior osteogenic differentiation potential upon cBM-MSCs in vitro. Schematic diagram of an in vitro osteogenic induction and the analyses of the osteogenic differentiation potential was showed (**A**). ALP activity at day 14 (**B**), matrix mineralization by *Von Kossa* staining with mineralized area percentage at day 7 and 14 (**C**), and osteogenic mRNA marker expression at day 7 and 14 (**D**) of cBM-MSCs and cDPSCs were investigated (n = 4). ALP activity was normalized with undifferentiated control. Relative mRNA expression was normalized with the reference gene, *Gapdh*, and undifferentiated control. Bars and asterisks indicated the significant difference comparing with control group (*p* value < 0.05).

cBM-MSCs and cDPSCs were able to differentiate toward osteogenic lineage in vitro, but in distinct potential as illustrated by the superior ALP activity at day 14 and ECM mineralization at day 7 and 14 of osteogenic cDPSCs (Fig. 2A–C). Further osteogenic mRNA marker analyses at day 7 and 14 illustrated that both cells showed trends of osteogenic marker expression in different magnitude. Osteogenic cBM-MSCs showed significant upregulation of *Ocn* at day 7 and *Osx* at day 14, while osteogenic cDPSCs revealed significant upregulation of *Runx2* and *Ocn* at day 7 and *Runx2*, *Alp*, *Ocn*, and *Osx* at day 14 (Fig. 2D). These findings suggested the superior osteogenic differentiation potential of cDPSCs upon cBM-MSCs in vitro.

### Different protein expression patterns upon an in vitro osteogenic differentiation by cBM-MSCs and cDPSCs

**Figure 3:**
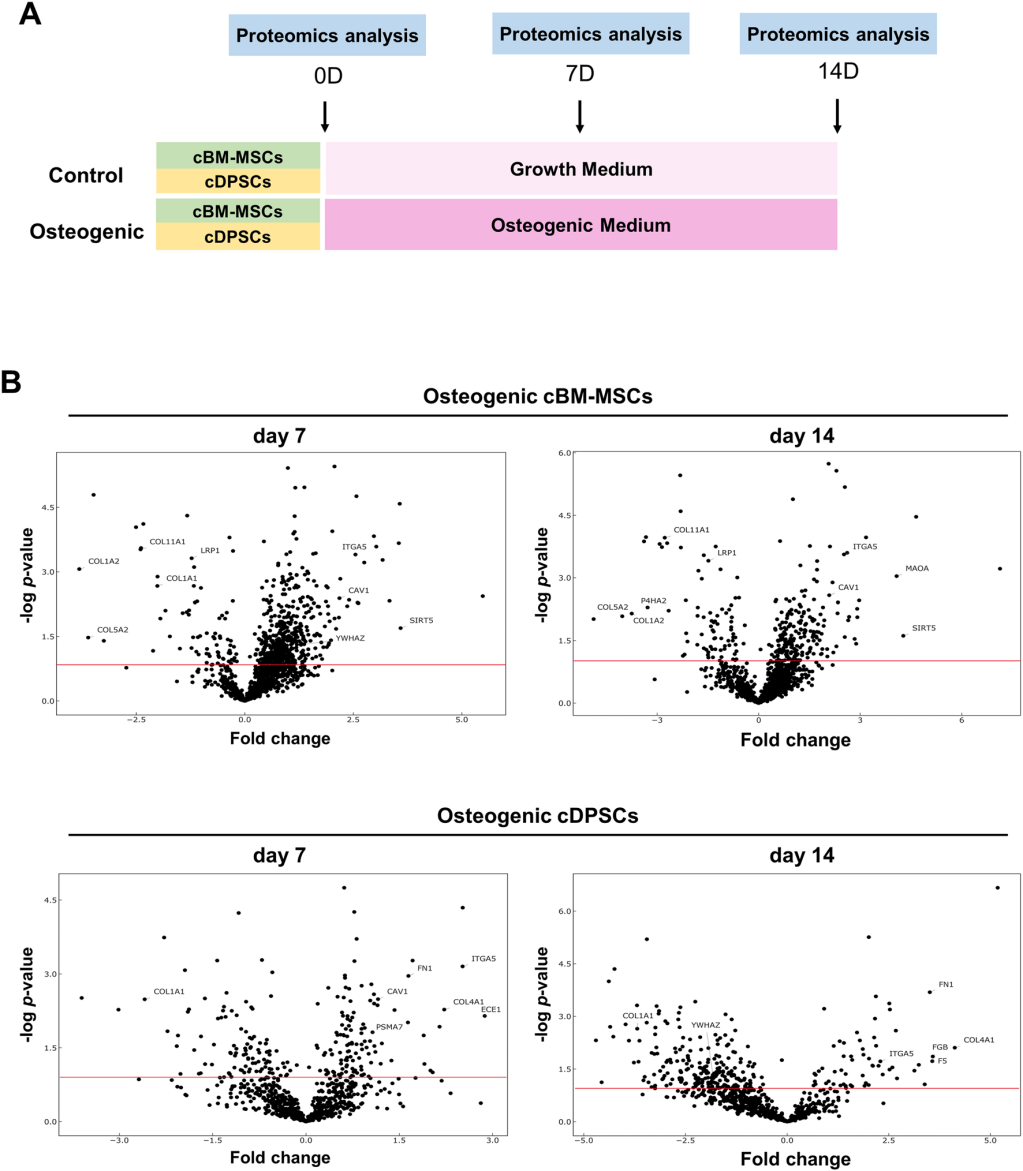
Different protein distribution patterns by cBM-MSCs and cDPSCs upon an in vitro osteogenic differentiation as assessed by Volcano plots. Schematic diagram of an in vitro osteogenic induction and the comparative proteomics-based systems biology analysis of the osteogenic differentiation behavior was showed (**A**). Volcano plots (n = 5) reflecting the distribution of expressed proteins by osteogenic cBM-MSCs and cDPSCs at day 7 and 14 post-induction were illustrated (**B**). The results were represented as − log *p* value and fold change (upregulation and downregulation). Red lines indicated *p* value < 0.05. Proteins located above the red line were significantly different compared with undifferentiated control (day 0).

**Figure 4:**
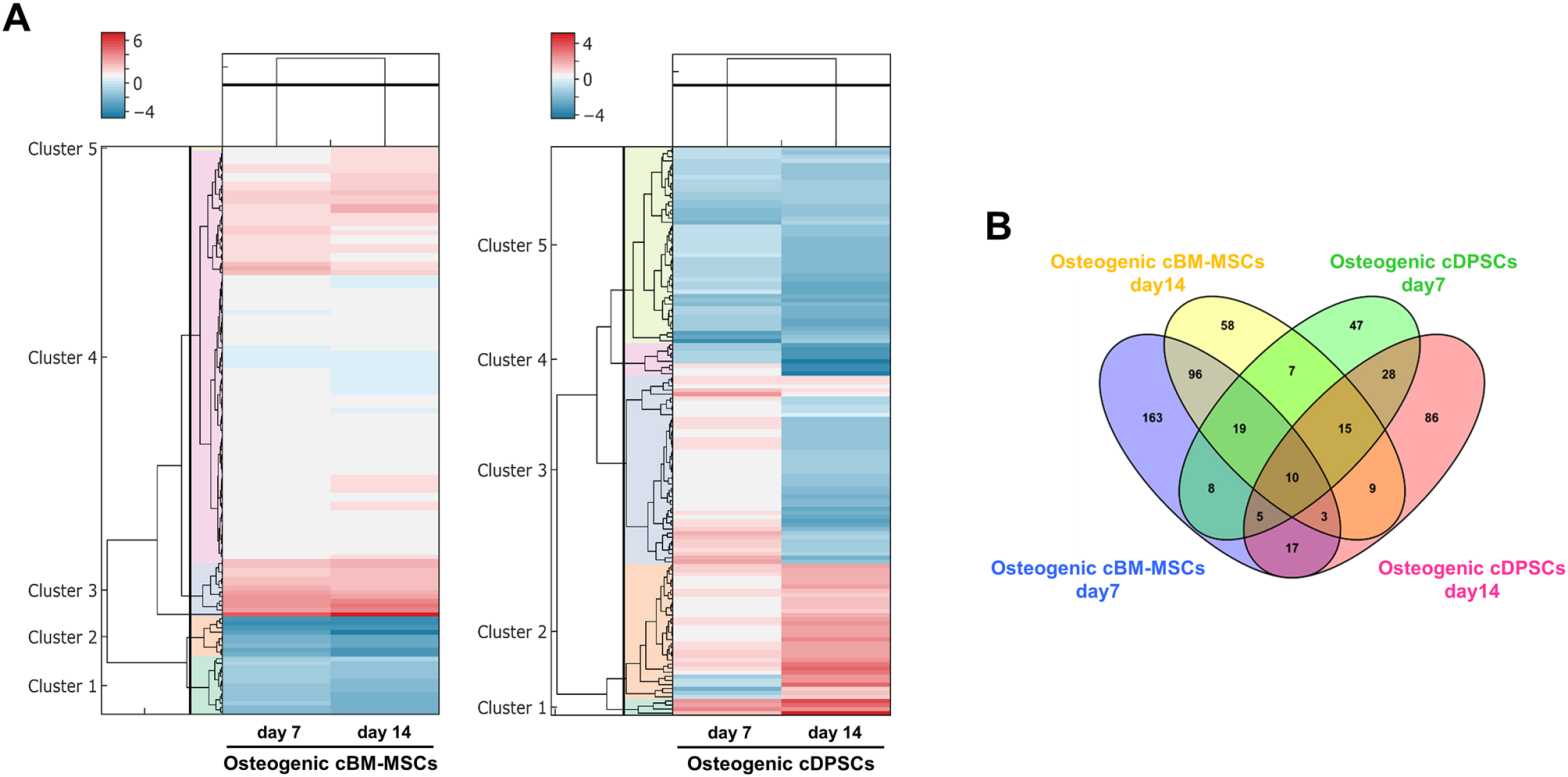
Different protein clustering patterns by cBM-MSCs and cDPSCs upon an in vitro osteogenic differentiation as assessed by heatmaps and Four-Circle Venn Diagram. Heatmaps with Dendrogram (n = 5) were illustrated for showing the clustering of significant expressed proteins by cBM-MSCs and cDPSCs upon an in vitro osteogenic differentiation at day 7 and 14 post-induction (n = 5). Color scale represents fold-change of protein upregulation (red) or downregulation (blue) (**A**). Four-Circle Venn Diagram (n = 5) illustrated the number of unique or overlapping proteins significantly expressed by cBM-MSCs and/or cDPSCs upon an in vitro osteogenic differentiation at day 7 and 14 post-induction (**B**).

Proteomics analysis and volcano plot at day 7 and 14 post osteogenic induction found the different protein expression pattern illustrating by upregulating trend in cBM-MSCs and slightly downregulating trend in cDPSCs (Fig. 3A,B). Further protein clustering on the heatmap provided 5 different clusters for particular cells with an interesting contrasting pattern between day 7 and 14 of cDPSCs which suggested a distinct underlying mechanism between them (Fig. 4A). Four-circle Venn diagram showed 359 and 201 identifiable proteins expressed by cBM-MSCs and cDPSCs, respectively (Fig. 4B). Interestingly, only 10 proteins were commonly co-expressed, but numerous proteins were uniquely expressed by each cell type (163 and 58 proteins by osteogenic cBM-MSCs, and 47 and 86 proteins by osteogenic cDPSCs, at day 7 and 14, respectively). This suggested a distinct protein expression pattern by cBM-MSCs and cDPSCs at each specific timepoint during an in vitro osteogenic differentiation.

### Quantitative proteomics profiling of cBM-MSCs and cDPSCs upon an in vitro osteogenic differentiation

Quantitative proteomics profiling heatmaps of osteogenic cBM-MSCs and cDPSCs based on DAVID and Reactome analyses were categorized regarding their osteogenic relevance, focusing on (a) signaling pathways, (b) cellular components and processes, and (c) cellular metabolisms. Different osteogenic protein expression patterns between two cell types were emphasized.

#### Signaling pathways

##### Kinase signaling pathways

**Figure 5:**
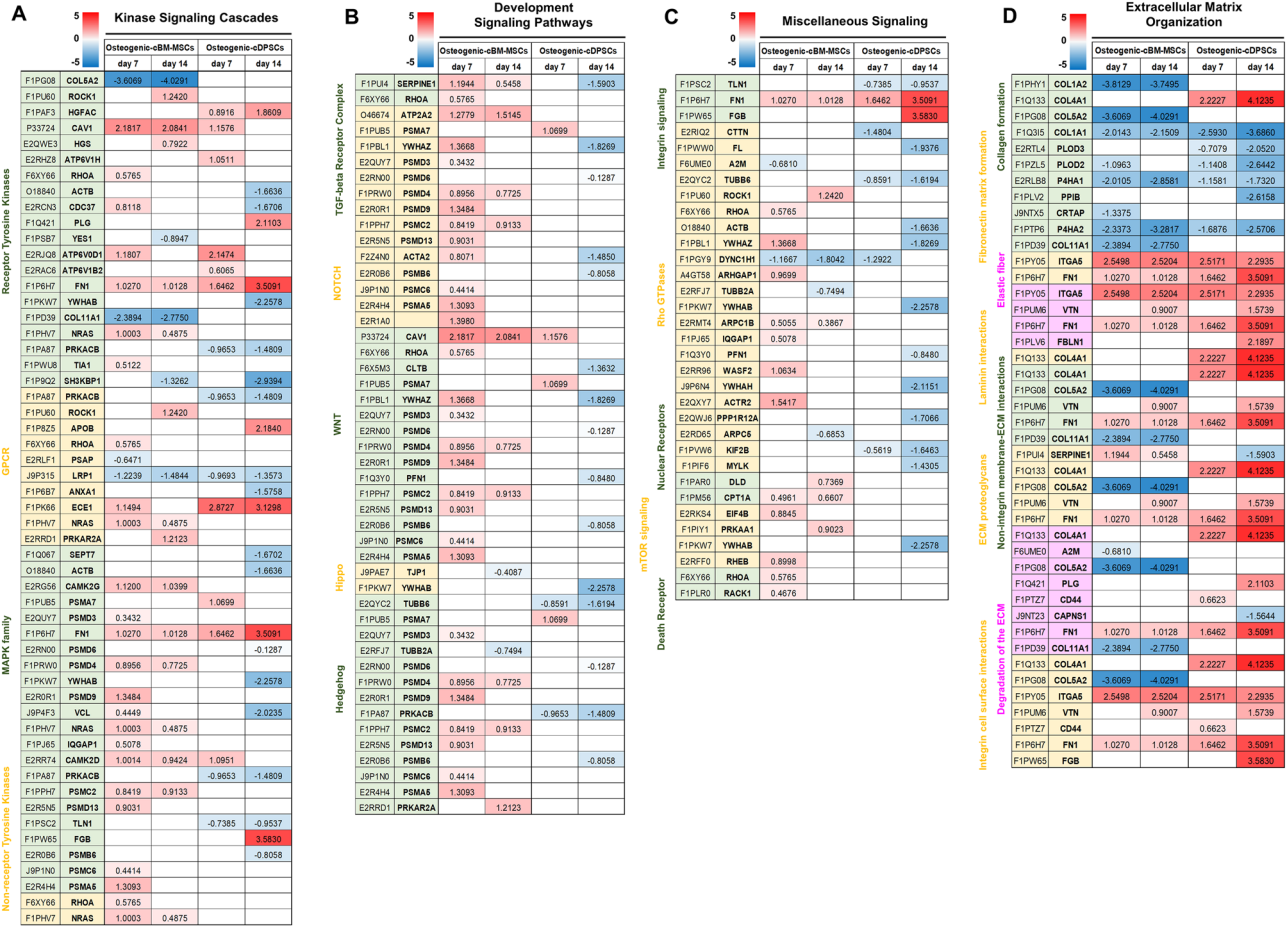
Unique quantitative proteomics profiling on signaling pathways and cellular components and processes by cBM-MSCs and cDPSCs upon an in vitro osteogenic differentiation. Quantitative proteomics profiling represented in heatmaps (n = 5) on signaling pathways and cellular components and processes were illustrated for providing a comparison of significant proteins expressed by cBM-MSCs and cDPSCs upon an in vitro osteogenic differentiation at day 7 and 14 post-induction. Intensifying color scale represented fold-change number of protein upregulation (red) or downregulation (blue). Proteins in categories of kinase signaling cascades (**A**), development signaling pathways (**B**), miscellaneous signaling (**C**), and extracellular matrix organization (**D**) were illustrated. Protein subcategories, protein names, and gene names were mentioned.

Analyses were focused on receptor tyrosine kinases (RTKs), G-protein-coupled receptors (GPCRs), mitogen-activated protein kinase (MAPK) family, and non-receptor tyrosine kinases (non-RTKs) (Fig. 5A). For RTK-related proteins, 2 main proteins regulating extracellular matrix (ECM) (COL5A2 and COL11A1) were downregulated in osteogenic cBM-MSCs but not in cDPSCs, while CAV1, ATP6V0D1, and FN1 which are considered as essential osteogenic proteins showed a relevance expression pattern in both cells. For GPCR-related proteins, ECE1 and APOB that are involved in the activation of osteoblastic proliferation^15^ were strongly upregulated in cDPSCs, whereas cBM-MSCs seemed rely on osteogenic regulators, ROCK1 and NRAS. For MAPK family and non-RTKs, it illustrated that cBM-MSCs mostly relied on the upregulation of these proteins, while cDPSCs tended to downregulate them. Most of upregulated members were proteasome proteins relating to an osteogenic differentiation (PSMA7, PSMD4, PSMD9, PSMC2, PSMD13, and PSMA5).

##### Development signaling pathways

Analyses were focused on five potential osteogenic signaling including TGF-beta receptor complex, Notch, Wnt, Hippo, and Hedgehog (Fig. 5B). The results showed an interesting trend of development signaling pathway-relating protein upregulation in cBM-MSCs, but not in cDPSCs. Most of the proteins were considered as the potential osteogenic regulators, suggesting different and unique underlying signaling pathways that govern osteogenic potential of the cells.

##### Miscellaneous signaling

Further analyses were pointed onto integrin signaling, Rho GTPases, nuclear receptor, mTOR signaling, and death receptor (Fig. 5C). The results indicated trend of protein upregulation in all signaling by cBM-MSCs, but not by cDPSCs which suggested contrasting roles of these signaling during osteogenic induction. In this context, some integrin-related proteins were upregulated by both cells, especially FN1 and FGB, suggesting a common pathway for ECM formation and mineralization^16,17^.

#### Cellular components and processes

##### ECM organization

Analyses were focused on collagen formation, fibronectin matrix formation, elastic fiber, laminin interactions, non-integrin membrane-ECM interactions, ECM proteoglycans, degradation of the ECM, and integrin cell surface interactions (Fig. 5D). The results illustrated that cBM-MSCs and cDPSCs showed quite similar expression profiles of proteins involving collagen formation, fibronectin matrix formation, and elastic fiber, while contrasting trends were found in other categories. Interestingly, COL4A1, a basement membrane component, was strongly upregulated by cDPSCs, but not cBM-MSCs.

##### Cell cycle

**Figure 6:**
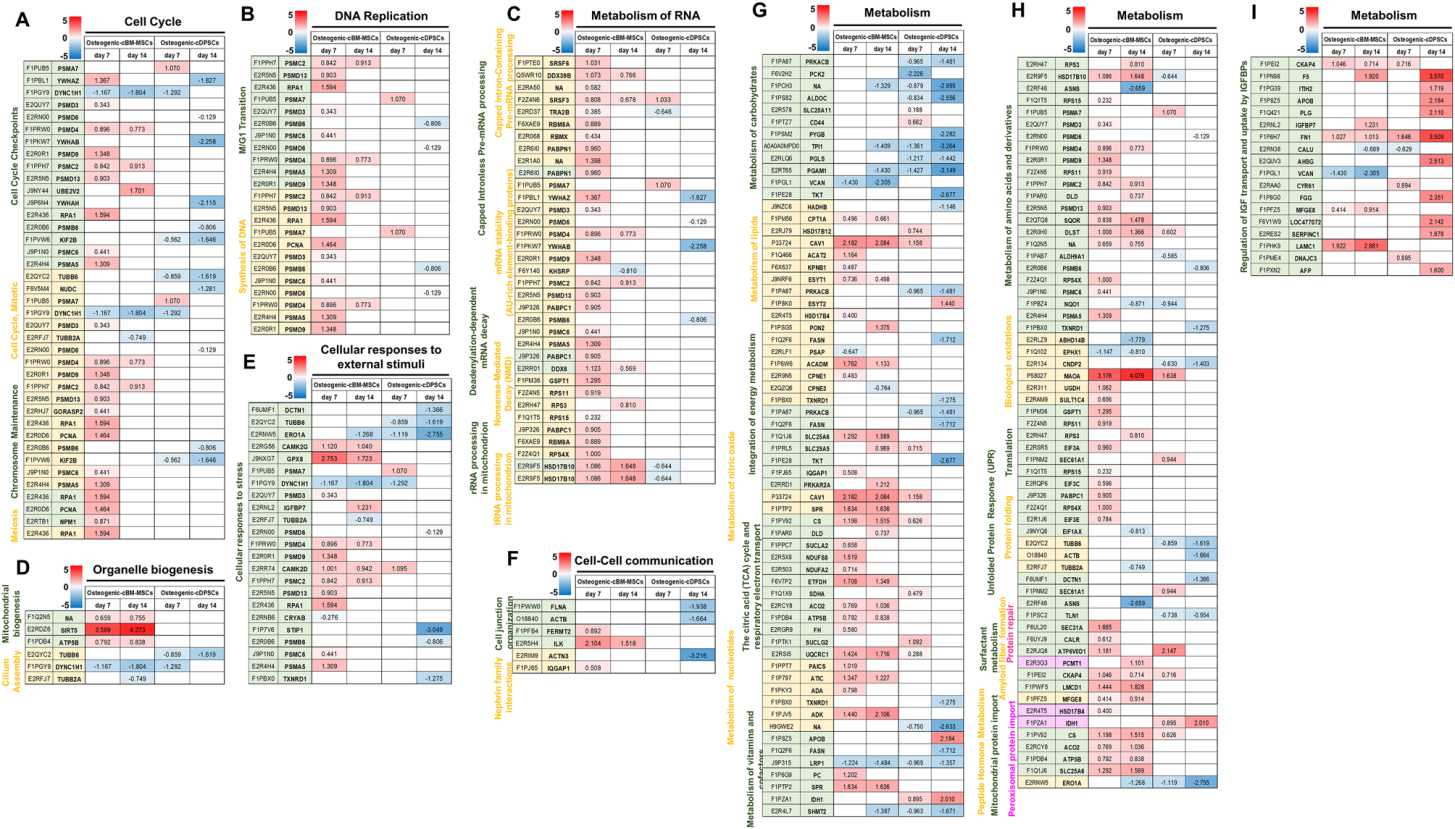
Unique quantitative proteomics profiling on cellular components and processes and cellular metabolisms by cBM-MSCs and cDPSCs upon an in vitro osteogenic differentiation. Quantitative proteomics profiling represented in heatmaps (n = 5) on cellular components and processes and cellular metabolisms were illustrated for providing a comparison of significant proteins expressed by cBM-MSCs and cDPSCs upon an in vitro osteogenic differentiation at day 7 and 14 post-induction. Intensifying color scale represented fold-change number of protein upregulation (red) or downregulation (blue). Proteins in categories of cell cycle (**A**), DNA replication (**B**), metabolism of RNA (**C**), organelle biogenesis (**D**), cellular responses to external stimuli (**E**), cell–cell communication (**F**), and cellular metabolisms (**G**–**I**) were illustrated. Protein subcategories, protein names, and gene names were mentioned.

Analyses were focused on cell cycle checkpoints, mitotic cell cycle, chromosome maintenance, and meiosis (Fig. 6A). Contrasting trends of protein expression in all categories were found. It seemed cDPSCs downregulated most of cell cycle-related proteins that might suggest the stage of cell differentiation.

##### DNA replication

Analyses were focused on M/G1 transition and synthesis of DNA (Fig. 6B). Trends of DNA-replication-related protein upregulation were markedly found in cBM-MSCs. These might suggest an active DNA replication of cBM-MSCs, especially for proliferating cell nuclear antigen (PCNA) which is used as an indicator of proliferative activity of neoplasm and osteoblast^18,19^.

##### Metabolism of RNA

Analyses were focused on capped intron-containing pre-mRNA processing, capped intronless pre-mRNA processing, mRNA stability (AU-rich element-binding proteins), deadenylation-dependent mRNA decay, nonsense-mediated decay (NMD), rRNA processing in mitochondrion, and tRNA processing in mitochondrion (Fig. 6C). Trends of upregulation were found in cBM-MSCs in all categories, but not in cDPSCs. These might suggest an active RNA metabolism by cBM-MSCs.

##### Organelle biogenesis

Analyses were focused on mitochondrial biogenesis and cilium assembly (Fig. 6D). It was showed that proteins involving mitochondrial biogenesis were upregulated by cBM-MSCs especially for SIRT5, whereas cilium assembly-related proteins were downregulated by both cBM-MSCs and cDPSCs.

##### Cellular responses to external stimuli

Analysis was focused on cellular responses to stress (Fig. 6E). The results illustrated the component of proteins that differently expressed by each cell which suggested a distinct response behavior between cBM-MSCs and cDPSCs.

##### Cell–cell communication

Analyses were focused on cell junction organization and nephrin family interactions (Fig. 6F). Contrasting trends were found in both categories suggesting an active cell–cell communication in cBM-MSCs, particularly for ILK which involves in osteoblast activity and mineralization^20^.

##### Cellular metabolisms

Analyses were focused on metabolism of carbohydrates, metabolism of lipids, integration of energy metabolism, metabolism of nitric oxide, the citric acid (TCA) cycle and respiratory electron transport, metabolism of nucleotides, metabolism of vitamins and cofactors, metabolism of amino acids and derivatives, biological oxidations, translation, protein folding, unfolded protein responses (UPR), protein repair, surfactant metabolism, amyloid fiber formation, peroxisomal protein import, and regulation of insulin-like growth factor (IGF) transport and uptake by insulin-like growth-factor binding proteins (IGFBPs) (Fig. 6G–I). It seemed that downregulating trend of metabolism of carbohydrate-related proteins and upregulating trend of proteins involving regulation of IGF transport and uptake by IGFBPs were found in both cBM-MSCs and cDPSCs. However, the rest of protein categories were contrastingly expressed by each cell type. Most of them were upregulated by cBM-MSCs, but not by cDPSCs suggesting a distinct cellular metabolic process during osteogenesis.

### Selection and confirmation of potential signaling underlying osteogenic differentiation by cBM-MSCs and cDPSCs in vitro

**Figure 7:**
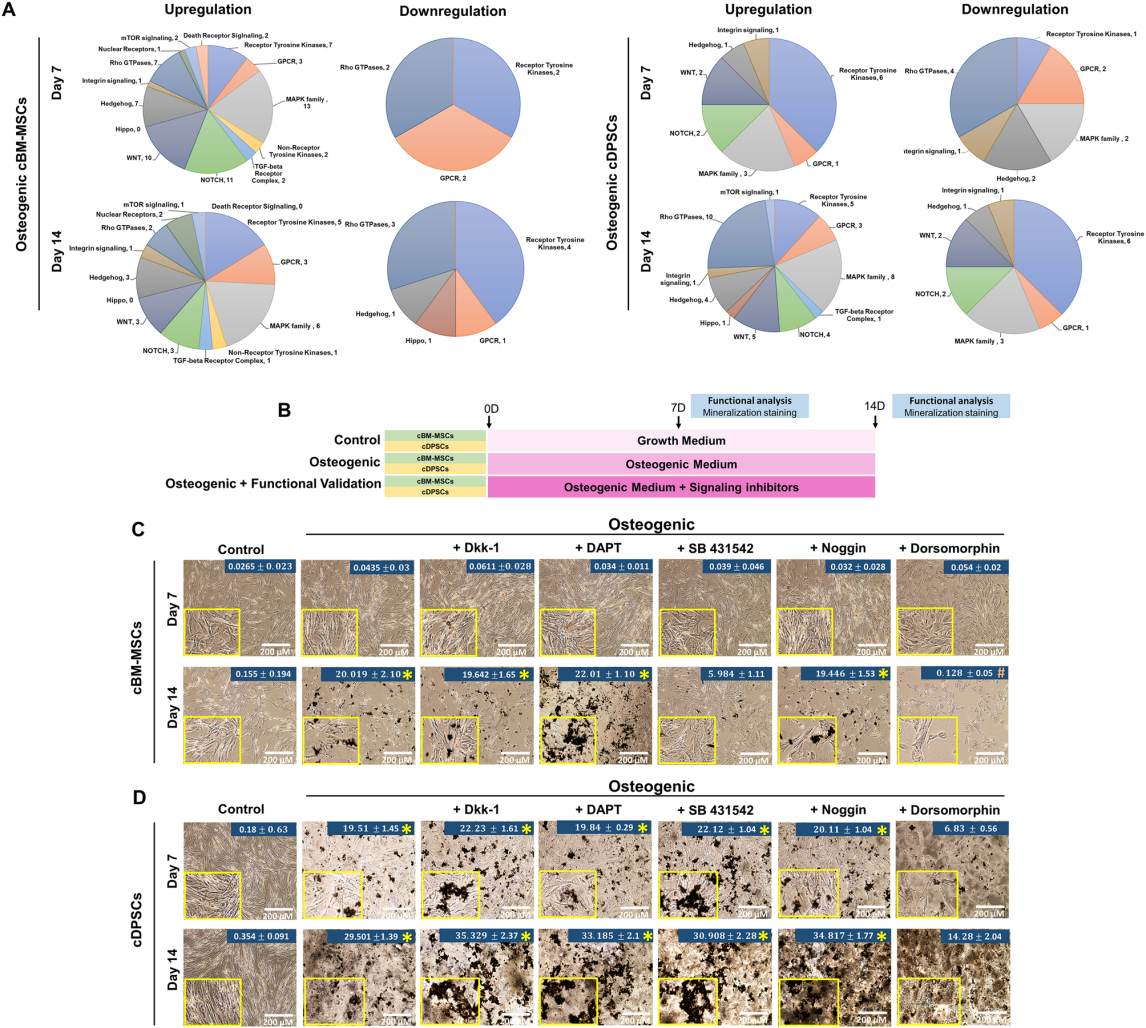
Trend of signaling protein expression and functional validation of selected potential signaling regulating osteogenic differentiation by cBM-MSCs and cDPSCs in vitro. Pie charts represented trend of signaling protein expression by cBM-MSCs and cDPSCs upon an in vitro osteogenic differentiation at day 7 and 14 post-induction (**A**). Signaling categories and numbers of protein were mentioned. Schematic diagram of an in vitro osteogenic induction and functional validation of selected potential signaling on osteogenic differentiation was showed (**B**). Functional validation of the potential signaling regulating an in vitro osteogenic differentiation by cBM-MSCs (**C**) and cDPSCs (**D**) was performed by treatment with specific signaling inhibitors (canonical Wnt inhibitor: Dkk-1, Notch inhibitor: DAPT, TGF-beta inhibitor: SB431542, and BMP inhibitors: noggin and dorsomorphin). Assessment of matrix mineralization by *Von Kossa* staining with mineralized area percentage was performed at day 7 and 14 post-induction (n = 4). Asterisks and sharps indicated the significant difference comparing with undifferentiated control and osteogenic control, respectively (*p* value < 0.05).

According to the quantitative proteomics analysis, trends of protein expression relating to signaling cascade during osteogenic differentiation by cBM-MSCs and cDPSCs were summarized (Fig. 7A). Set of potential signaling underlying an in vitro osteogenic differentiation were selected and functionally validated based on mid- and late-state ECM mineralization upon specific inhibitor treatment (Fig. 7B–D). cDPSCs showed a superior osteogenic differentiation potential upon cBM-MSCs at day 7 and 14 post-induction. Roles of Wnt signaling were analyzed using canonical Wnt inhibitor, Dkk-1, and the results showed markedly enhanced ECM mineralization by cDPSCs but not cBM-MSCs at day 7 and 14, suggesting a contrasting role of canonical Wnt in this regard. Notch signaling was further studied using gamma secretase inhibitor (GSI), DAPT, and the results illustrated the dramatic enhancement of ECM mineralization by both cells at day 14, reflecting the late-state benefit of Notch inhibition. Transforming growth factor (TGF)-beta was additionally validated using SB431542, a TGF-beta type I receptor blocker, and the results showed attenuated ECM mineralization by cBM-MSCs but strong enhancing effect in cDPSCs at day 7 and 14. These suggested contrasting effects of TGF-beta/activin/NODAL pathway manipulation on mid- and late-state ECM mineralization. Bone morphogenetic protein (BMP) was validated using two different antagonists, noggin and dorsomorphin. Interfering by endogenous BMP antagonist, noggin, resulted in enhanced ECM mineralization by cDPSCs but not cBM-MSCs at day 14, while BMP type I receptor inhibitor, dorsomorphin, suppressed ECM mineralization by both cells at day 7 and 14, suggesting a complex role of BMP during osteogenesis. Thus, the selected signaling cascades derived from quantitative proteomics analysis were the potential pathways underlying osteogenic differentiation by cBM-MSCs and cDPSCs.

### Tree diagram-based comprehensive analysis of selected potential signaling regulating osteogenic differentiation by cBM-MSCs and cDPSCs in vitro

**Figure 8:**
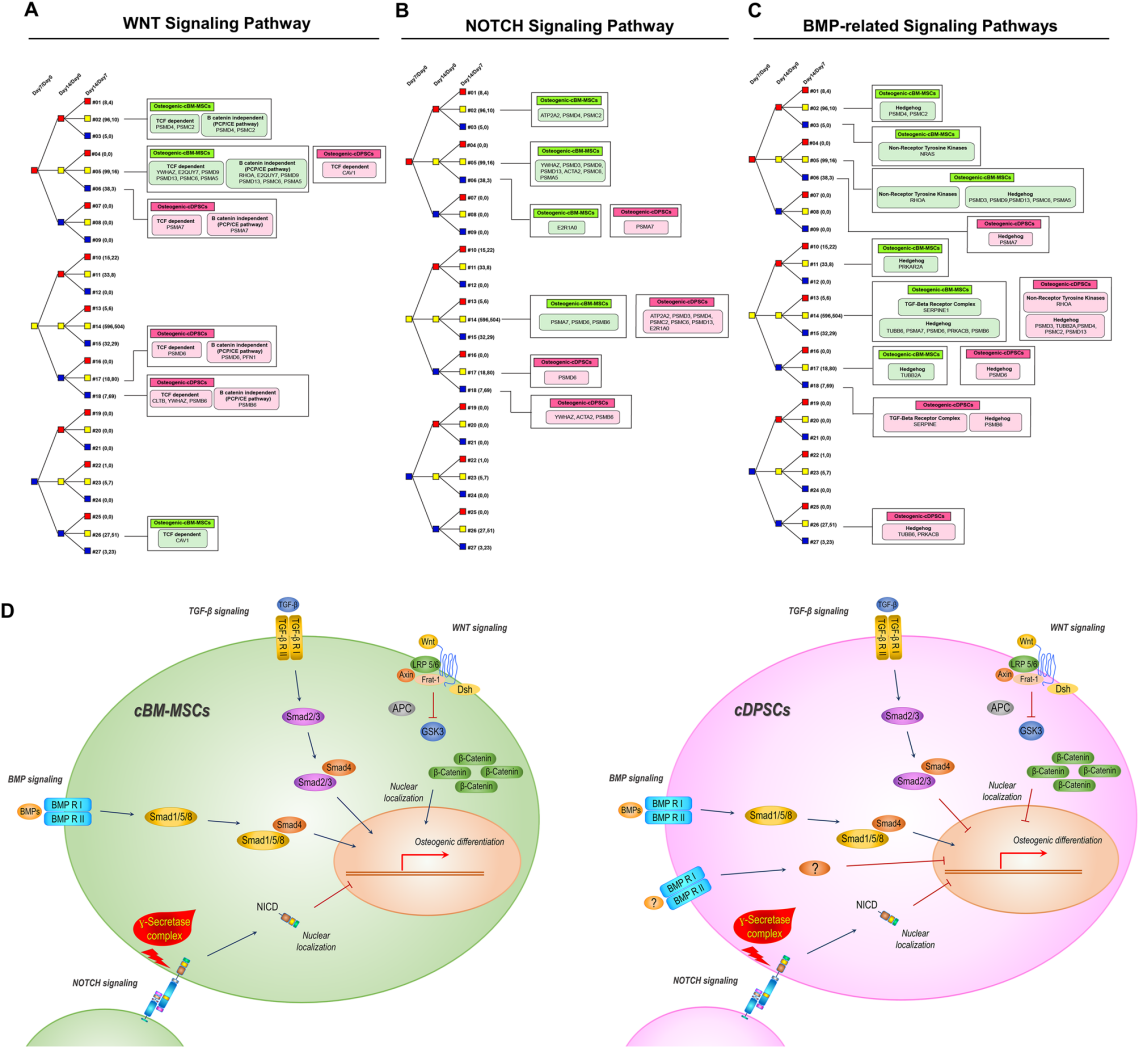
Tree diagram-based comprehensive analysis and pathway summary of selected potential signaling regulating osteogenic differentiation by cBM-MSCs and cDPSCs in vitro. For comprehensive understanding on signaling dynamic, tree diagram analysis of functional validated signaling was performed by tracking protein relationship at 3 timepoints during osteogenic differentiation (day 7 vs. 0, day 14 vs. 0, and day 14 vs. 7), comprising Wnt signaling pathway (**A**), Notch signaling pathway (**B**), and BMP-related signaling pathways (**C**). The diagrams illustrated dynamic changing of signaling components in cBM-MSCs (green box) and cDPSCs (pink box), while tree diagram connectors represented significant protein upregulation or downregulation by red or blue code, respectively. Yellow code represented unchanged protein level. All episodes were coded as #1 to #27. Numbers in the brackets referred to total protein numbers respectively expressed by osteogenic cBM-MSCs and cDPSCs in each episode. Summary of unique signaling relationship on osteogenic differentiation by cBM-MSCs and cDPSCs was illustrated (**D**).

For comprehensive understanding on signaling dynamic, tree diagram analysis of 3 functional validated signaling was performed by tracking protein relationship at 3 timepoints during osteogenic differentiation (day 7 vs. 0, day 14 vs. 0, and day 14 vs. 7) (Fig. 8A–C). Wnt-related proteins were analyzed as (1) T-cell factor (TCF) dependent pathway or canonical pathway and (2) beta-catenin independent pathway (planar cell polarity (PCP)/convergent extension pathway) or non-canonical pathway. It showed that cDPSCs downregulated most components of TCF dependent and beta-catenin independent pathways at day 14, while cBM-MSCs upregulated and maintained those components since day 7. Notably, CAV1 acted contrastingly in both cells (Fig. 8A). Notch analysis found that cDPSCs tended to suppress Notch components at day 14, but cBM-MSCs kept upregulating and maintaining them from day 7 (Fig. 8B). BMP-related pathways were further analyzed as (1) TGF-beta receptor complex, (2) non-receptor tyrosine kinase (non-RTK), and (3) hedgehog. It found that cDPSCs tended to suppress components of TGF-beta receptor complex and hedgehog, especially at day 14, and seemed not relying on non-RTK. Contrastingly, cBM-MSCs seemed relying on upregulating components of hedgehog and non-RTK, obviously since day 7, while those in TGF-beta receptor complex were kept unchanged (Fig. 8C). The summary of unique signaling relationship on osteogenic differentiation by cBM-MSCs and cDPSCs was illustrated in Fig. 8D.

## Discussion

According to the correction of skeletal and maxillofacial bone defects in veterinary practice, trend of stem cell-based regenerative treatment is of interest, especially for the application of BTE^9^. In this regard, the potential stem cell resources for the generation of bone-forming cells are the crucial factors determining the success of the treatment. We found that the potential cMSC resources for canine BTE were cBM-MSCs and cDPSCs, due to their plasticity, availability, and accessibility^4,21^. From our preliminary study and published report, cMSCs behaved differently from human MSCs (hMSCs) regarding the osteogenic differentiation potential (in-house data)^8,9^. We found that cMSCs showed matrix mineralization earlier than hMSCs did, so the osteogenic differentiation process and underlying mechanism might be different between cMSCs and hMSCs. From this reason, we cannot imply the osteogenic differentiation knowledge of hMSCs directly to the cMSC application.

To address the potential problem of cBM-MSCs and cDPSCs for the in vivo autogenic transplantation, regarding the invasive procedure for cBM-MSC collection and the limited volume of pulp tissue by pulpectomy or pulpotomy for cDPSC isolation, previous study has reported the successful in vivo experiment showing the opportunity for allogenic transplantation^22^. However, the lack of global data of osteogenic differentiation behavior of canine MSCs resulting in the delay to modify bone induction process for transplantation toward clinical use.

Here, the isolated cBM-MSCs and cDPSCs presented the stemness property, typical mesenchymal stem cell morphology, adherent property, colony-forming characteristic, and multi-lineage differentiation potential regarding adipogenicity, chondrogenicity, and osteogenicity. Each population of cBM-MSCs and cDPSCs in the present study has been postulated to be MSCs by International Society for Cellular Therapy (ISCT) guideline^23^. Positive CD73 and CD90 expression were markers of MSC characteristic following ISCT requirement, however, the surface marker expression of CD73 was low in cBM-MSCs and cDPSCs. Our results correlated tested CD73 from feline and horse MSCs that showed low expression of CD73^24,25^. It should be suggested that CD73 expression in animal MSCs may be different from hMSCs. Therefore, the phenotype of MSCs should be more investigated in different sources and species.

In this study, an in vitro osteogenic differentiation by cBM-MSCs and cDPSCs was thoroughly explored, and we found that both cells contained a different osteogenic differentiation potential and unique cellular behavior during differentiation process. It seemed that cDPSCs possessed a superior osteogenic differentiation potential upon cBM-MSCs regarding ALP activity, ECM mineralization, and osteogenic marker expression. When compared the results between cDPSCs and cBM-MSCs at day 7 and 14, osteogenic cDPSCs showed the promising phenotype (ALP activity and matrix mineralization) and genotype (osteogenic mRNA marker expression) comparing with osteogenic cBM-MSCs. In addition, previous publication presented that the capacity of osteogenic differentiation of DPSCs was higher than BM-MSCs^22^. Therefore, cDPSCs indicated superior osteogenic capacity in vitro.

This present results have been supported by cMSC research comparing between BM-MSCs and DPSCs according to osteogenic differentiation potential both in vitro and in vivo^26,27^. It is interesting that hMSCs derived from bone marrow, synovial fluid, adult dental pulp, and exfoliated deciduous teeth showed distinct characteristics on osteogenic, chondrogenic, adipogenic, and neurogenic differentiation potentials. It has been suggested that hBM-MSCs indicated higher level of osteogenic capacity than human DPSCs (hDPSCs)^28^. Therefore, different species of MSCs indicated the different potential of osteogenic differentiation.

Regarding the osteogenic mRNA marker profiles of the osteogenic cBM-MSCs and cDPSCs, it should be noted that both cells showed trends of osteogenic marker expression in different magnitude which might due to the distinct oscillation of the osteogenic differentiation stage by each cell type. Osx is crucial transcription factor for differentiation participating in the conversion of premature into mature osteoblasts, the upregulation of Osx is an applicable marker of final step into osteogenic lineage. Our result illustrated the *Osx* upregulation in osteogenic cBM-MSCs at day 14. This trend is similar result from previous publication^29^. Therefore, it could be implied for final bone induction at day14. In addition, Osx is a late-stage transcription factor, while Alp and Runx2 are early-stage transcription factors of osteogenesis. It is possible that cBM-MSCs were induced toward osteogenesis earlier than those from human which showed upregulation of early stage marker. This phenomenon could also impact hMSCs to generally observe bone induction at day 21, whereas cMSCs were considered at day 14. Previous study has also established osteogenic cBM-MSCs at day 14^8,30^. Therefore, due to previous reports and our preliminary study that showed the upregulation of late state osteogenic gene and mineralization, cMSCs were induced with osteogenic medium and indicated the result at day 14.

Previous studies mostly focused on individual osteogenic differentiation potential of the cells, rather than comparing and clarifying their osteogenic differentiation behavior^8,9,31,32^. To address this concern, comparative proteomics-based systems biology analysis was performed for dissecting the osteogenic differentiation behavior of cBM-MSCs and cDPSCs in vitro*,* since quantitative proteomics analysis has been proposed as a promising tool for comprehensively analyzing an osteogenic differentiation by many MSC-based osteogenic models^33,34^. Time-series analysis (day 0 vs. day 7 vs. day 14) of osteogenic differentiation behavior by cBM-MSCs and cDPSCs was conducted to thoroughly clarify this issue. The systems biology analytic approach and functional validation assay were used for dissecting the osteogenic differentiation behavior and crucial underlying mechanisms governing their osteogenic differentiation potential. Here, we successfully used a dimethyl labelling with liquid chromatography/mass spectrometry (LC/MS/MS)-based peptide sequencing to selectively label, purify, and identify proteins from osteogenic cBM-MSCs and cDPSCs at day 7 and 14 post-induction, then compared to undifferentiated control following previous protocol^35^. By this method, we were able to analyze all samples from each cMSC at the same time to reduce analytical variation. Besides, we set the strong selection criteria for including the proteins into the lists of potential signaling and further validation assay. To avoid the biased selection of protein, we calculated the protein expression level as Log2 normalized ratio at day 7 and 14, comparing with undifferentiated control at day 0. The relevant proteins found less than 3 from 5 replicates were excluded. Then, the mean and standard deviation of fold change across all 5 biological replicates were determined. By this protocol, we obtained the high significant protein lists and their relative expression levels as illustrated in Figs. 3 and 4. Then, the listed proteins and underlying mechanisms were double checked with our in-house data and previous publications before the validation assay. The tree diagrams and illustrations in Fig. 8 were summarized according to the results from proteomics analysis and validation assay.

To explore the protein expression pattern, protein allocation was plotted using volcano plot, heatmap clustering, and Four-Circle Venn Diagram, and the results showed that osteogenic cBM-MSCs and cDPSCs had a unique protein expression pattern as seen in different protein expression distribution in volcano plot and heatmap clustering. Four-Circle Venn Diagram also showed that 163 and 58 proteins were uniquely expressed by cBM-MSCs, while 47 and 86 proteins were only found in cDPSCs, at day 7 and 14 respectively. Previous proteomics analysis of human osteoarthritis patients identified the upregulating trend of complement proteins^36^. Additionally, 1,943, 2,084, and 2,274 of human BM-MSC total proteins were found from quantitative phosphoproteomics profiling at day 1, 3, and 7 of osteogenic induction^37^. These suggested an importance of comprehensive analysis on particular disease or osteogenic model since the expression patterns were varied.

In this study, the quantitative proteomics profiles were then mapped and annotated with the online database, Reactome and DAVID, in order to categorize the expressed proteins based on their cellular and physiological functions. In this regard, heatmaps were used, and the proteins were categorized into 3 main groups: (a) signaling pathways, (b) cellular components and processes, and (c) cellular metabolisms. According to the unique expression patterns seen in volcano plot, heatmap clustering, and Four-Circle Venn Diagram, we decided to focus on the set of functional proteins that showed distinct expression pattern between osteogenic cBM-MSCs and cDPSCs. Interestingly, most of the annotated proteins in 3 categories showed different expression levels between 2 cell types, and the expression patterns were very dynamic during the osteogenic induction process which suggested the distinct underlying mechanisms and potential functional proteins/components for controlling their osteogenicity. These findings were correlated with the osteogenic behavior of several MSCs derived from different sources that uniquely contained their osteogenicity and underlying differentiation processes/pathways^34,38–40^.

As the most potential osteogenic regulators are the underlying signaling pathways that enhance or suppress osteogenic differentiation processes and other cellular activities resulting in different osteogenic, osteoinductive, and osteoconductive properties^41,42^, we thoroughly dissected the osteogenic signaling components and found 4 potential osteogenic regulating cascades including Wnt, Notch, TGF-beta, and BMP signaling pathways. These identifications were also related to previous reports suggesting the pivotal roles of the selected pathways on osteogenicity of various cells and osteogenic models^43–46^. It was quite surprise that functional validating assays of the selected signaling pathways showed the contrasting effects upon specific inhibitor treatments between 2 cell types which indicated the unique and distinct roles of osteogenic governing pathways for each cell type. These findings emphasized the influences of signaling pathways underlying the variety of cell and tissue osteogenicity mentioned in many reports^47–49^. Integrating with tree diagram-based comprehensive analysis, we found in this study that cBM-MSCs required the presences of Wnt (canonical), TGF-beta, and BMP signaling, while cDPSCs mainly relied on the BMP signaling presentation during osteogenic differentiation in vitro. These correlated to the findings in human and mouse embryogenesis that Wnt, TGF-beta, and BMP signaling played the beneficial roles on osteoblastic differentiation and osteogenesis^42,50,51^. The importance of BMP signaling have widely been recognized as a promoter of bone formation and might be a potential bone-promoting molecule for bone regeneration^52^. However, we found the contrasting effects of 2 BMP antagonists used in this study. Noggin and dorsomorphin have been reported to negatively regulate BMP activities during osteogenesis^53,54^. Noggin binds to BMPs with high affinity and blocks BMPs’ binding to the BMP receptor, while dorsomorphin inhibits Smad activation, a downstream pathway of BMP signaling^54,55^. We found that dorsomorphin completely inhibited osteogenic differentiation by cBM-MSCs and showed partially effect in cDPSCs. These findings suggested that BMP signaling via Smad-dependent cascade might be the principle pathway of cBM-MSCs and cDPSCs for differentiating toward osteogenic lineage. In contrast, noggin treatment showed a beneficial effect on osteogenic differentiation by cDPSCs, but not in cBM-MSCs. Recent study showed that noggin significantly increased ALP activity and simplified osteogenic differentiation^56^. Our results might reflect a diverse role of BMP ligands on osteogenic differentiation by cDPSCs which requires further clarification.

It seemed that, during osteogenic differentiation, Notch signaling tended to be downregulated in order to suppress cell proliferation and trigger Wnt cascade for further osteogenic differentiation process^51,57^. These correlated to our findings that canonical Notch signaling inhibitor could enhance osteogenic differentiation by both cBM-MSCs and cDPSCs. It was interesting that the responses on specific Wnt or TGF-beta blocking were contrast between the cells. It seemed that cBM-MSCs tended to upregulate Wnt and TGF-beta signaling components, and their osteogenic differentiation potential relied on the presences of these signaling pathways. Many publications have also reported the beneficial roles of Wnt and TGF-beta on the osteogenic differentiation by various MSCs^47,58,59^. In contrast, cDPSCs managed to have Wnt and TGF-beta signaling components downregulated, and it seemed that osteogenic differentiation of the cells was not mainly relied on the presences of these signaling cascades. We also found that treatment with specific Wnt or TGF-beta inhibitor gave beneficial results on osteogenic differentiation by cDPSCs, suggesting a contrasted role of Wnt and TGF-beta on osteogenic differentiation controlling between 2 cell types.

In proteomics experiment, we earned the protein list and dynamic protein expression from database analysis. Moreover, database analysis showed the potential signaling that should be related in osteogenic differentiation of cBM-MSCS and cDPSCs. However, we could not know the function of protein by proteomics experiment. To further using in clinical application, we decided to select the main osteogenic pathway to explore and analyze. So, inhibitor experiment was represented as functional analysis to confirm function of selected pathway. To select inhibitor, the specific canonical inhibitor for each signaling was chosen to get a confirmation result of each signaling after blocking of two cells. For example, Dkk-1 is generally used as an inhibitor of canonical Wnt signaling or SB431542 is acceptable inhibitor to block TGF-beta signaling. In this regard, Fig. 8 were summarized from candidate pathways that were chosen from proteomics analysis together with inhibitor results that showed the relevant of functional pathways deal with osteogenic differentiation of cBM-MSCs and cDPSCs.

Beside the comprehensive analysis and functional validation of the potential signaling pathway regulating osteogenic differentiation of the cells, we found the expressions of various potential proteins in other categories that might be the major components manipulating osteogenic differentiation due to previous supporting reports. In eukaryotic cells, the proteasome (PSM) is a complex molecule constructed from large proteins, namely proteasome endopeptidase complex subunits, and relates to ubiquitin pathway which is the mechanisms controlling intracellular proteolysis^60^. Previous research showed that ubiquitin–proteasome pathway involved in osteogenesis both in vitro and in vivo^61–63^. It is suggested that inhibition of the proteome process by specific inhibitors could enhance bone formation by an activation of BMP-2 expression^64^. In this study, we found that proteins in PSM family were dynamically expressed in both cBM-MSCs and cDPSCs and closely related to set of proteins relating MAPK family as well as Notch, Wnt, and hedgehog signaling. Further study on PSM family protein might clarify their contribution on osteogenicity of the cells.

It should be suggested that cBM-MSCs and cDPSCs mostly employed the different set of proteasome-related proteins. Currently, proteasome become more interesting since previous study showed that it is the crucial protein which involved in Wnt canonical pathway to breakdown a key protein called β-catenin for osteoblasts. This process showed the importance of proteasome-mediated β-catenin degradation in osteoblast and osteoclast development. Moreover, there is evidence that proteasome may be an effective molecule to involving in Notch signaling^65^, TGF-beta and BMP signaling^66^. While Mass spectrometry technique and database is still going on to develop the efficient method to get the global protein analysis for animal, the detection of other component proteins such as proteasome could use to predict the relevant signaling. It is certain that only one proteasome could not indicate the specific signaling. The abundant of proteasome result can use to show the relevant signaling and help us to more consider of that pathway. When the proteomics results had been identified, we employed functional validation analysis by using canonical inhibitor to confirm the proteomics results concentrating on osteogenic signaling of cBM-MSCs and cDPSCs. Therefore, every protein was valuable to suggest relevant behavior of cBM-MSCs and cDPSCs toward osteogenesis, and that was put into the tree diagram in each involving signaling.

Based on cellular component and process analyses, it has been reported that collagen type I alpha 1 (COL1A1) and collagen type I alpha 2 (COL1A2) are proteins which support bone tissues, and mutations of COL1A1 and COL1A2 are related to osteogenesis imperfecta^67^. However, they were downregulated in cBM-MSCs and cDPSCs during an osteogenic induction, while integrin subunit alpha 5 (ITGA5), fibronectin 1 (FN1), and vitronectin (VTN) were upregulated. ITGA5 promotes osteoblastic differentiation in human MSCs by increasing Runx2 expression and activity^68^. VTN is a multifunctional glycoprotein found and involved in various physiological processes and promotes cell attachment in bone and ECM^69^. Though the expression of collagen type IV alpha 1 (COL4A1) suggests an underlying molecular mechanisms for osteopenia^70^, the results revealed that COL4A1 expression in osteogenic cDPSCs were principally upregulated. Thus, further experiments are necessary to address the function and the difference of ECM organization between osteogenic cBM-MSCs and cDPSCs.

Further analysis revealed that several proteins of osteogenic cBM-MSCs and cDPSCs involved with metabolism of carbohydrate were downregulated, while several proteins in metabolism of lipids were upregulated. Previous study suggested that bone mineral density rises with body fat mass, and obesity has a protective effect against osteoporosis^71^. However, recent study from rat bone marrow found that low-carbohydrate with high-fat diets have negative influence during osteogenesis by reducing osteogenic transcription factors (*Runx2*, *Osterix*, and *C/EBPβ*)^72^. This indicates that osteogenesis from different cell sources may employ different metabolism for bone formation or resorption.

In addition to proteins in post-translational protein modification, recent studies have provided some evidence that IGFBP7, an insulin-like growth factor-binding protein 7, increases the osteogenic differentiation of BMSCs by the Wnt/β-catenin signaling pathway^73^. In addition, the presence of Ras homolog gene family member A, RHOA, indicated its involvement in cytoskeleton rearrangement of BM-MSCs^74,75^. These findings suggest that proteins in post-translational protein modification are required for cBM-MSCs and cDPSCs osteogenic differentiation. In addition, insulin-like growth factors, IGFs, play a role during fetal development and postnatal growth in several cell types^76^. Upregulated protein related to IGFs including IGFBP7 and FN1 can enhance osteogenesis of MSCs^77^. Therefore, cBM-MSCs and cDPSCs might utilize IGFs for osteogenic differentiation, which further requires study.

In conclusion, we used the comparative proteomics-based systems biology analysis for dissecting an in vitro osteogenic differentiation behavior by cBM-MSCs and cDPSCs and found that cDPSCs contained a superior osteogenic differentiation upon cBM-MSCs. Functional analysis along with hierarchical clustering for tracking protein dynamic change showed that cBM-MSCs required the presences of Wnt, TGF-beta, and BMP signaling, while cDPSCs mainly relied on the BMP signaling presentation during osteogenic differentiation. This comprehensive data will be a crucial information for further deep mechanism study and the establishment of cMSC-based BTE for veterinary practice.

## Methods

### Cell isolation, culture, and expansion

All protocols were conducted in accordance with guidelines and regulations approved by the Institutional Animal Care and Use Committee (IACUC), Faculty of Veterinary Science, Chulalongkorn University (Animal Use Protocol No.1531038). Each cell type was obtained from five healthy dogs aged 3–10 years old. Dental pulp tissue and bone marrow were collected from different dogs. cBM-MSCs were isolated according to previous published protocol^8^. Briefly, canine bone marrow was aspirated and washed twice with Hank’s Balanced Salt Solution (HBSS, Thermo Fisher Scientific, USA). The mixture was centrifuged at 300*g* for 15 min and 1,000*g* for 5 min, respectively, then pellet was gently resuspended and seeded onto T-75 culture flasks (Corning, USA). For cDPSC isolation, tissue explant technique was used^78^. Pulp tissue was collected with aseptic technique and placed onto tissue culture dishes (Corning, USA).

cBM-MSCs were maintained in Dulbecco’s Modified Eagle Medium/F12 (DMEM/F12) (Thermo Fisher Scientific, USA), while cDPSCs were cultured in DMEM. Both media were supplemented with 10% fetal bovine serum (FBS)  (Thermo Fisher Scientific, USA), 1% Glutamax (Thermo Fisher Scientific, USA), and 1% Antibiotic-Antimycotic (Thermo Fisher Scientific, USA). Cells were incubated in 5% CO_2_ and 95% air at 37 °C with every 48 h media substitution. Cells were subcultured when 80% confluence reached. Cells from passage 2–5 were used in the experiments.

### Reverse transcription, quantitative polymerase chain reaction (RT-qPCR)

Total cellular RNA was isolated using TRIzol-RNA isolation reagent (Thermo Fisher Scientific, USA) and DirectZol-RNA isolation kit (ZymoResearch, USA). The complementary DNA (cDNA) was obtained by using reverse transcriptase enzyme kit (Promega, USA). Quantitative real-time PCR (qPCR) was performed using FastStart-Essential DNA Green Master (Roche Diagnostics, USA) and Bio-rad Real-Time PCR Detection System (Bio-rad, USA). The mRNA expression was illustrated as relative mRNA expression by normalizing to *Gapdh* and control.

### Flow cytometry

To characterize MSC surface marker expression, single cell suspension was stained with mouse anti-CD73 monoclonal antibody (Thermo Fisher Scientific, USA) and FITC-conjugated goat anti-mouse immunoglobulin (Ig) G secondary antibody (Bio-rad, USA), PE-conjugated rat anti-CD90 monoclonal antibody (eBioscience, USA), and FITC-conjugated mouse anti-CD45 monoclonal antibody (BioLegend, USA). Mouse IgG2a kappa Isotype (BioLegend, USA), PE-conjugated rat IgG2b kappa Isotype (BioLegend, USA), and FITC-conjugated mouse IgG1 kappa Isotype (BioLegend, USA) were used as isotype control. The stained cells were analyzed using FACS caliber flow cytometer (BD biosciences, USA) (n = 4).

### Colony formation assay

The cells were seeded at 1,500 cells per 60 mm culture dish (Corning, USA). After 2 weeks, the cells were fixed with 100% methanol (Sigma-Aldrich, USA) and stained with crystal violet (Sigma-Aldrich, USA). Cell clump containing more than 50 aggregated cells was counted as a colony (n = 4).

### In vitro adipogenic differentiation

According to previous established protocol^78,79^, 30,000 cells were seeded onto 24-well plate (Corning, USA) and cultured in adipogenic induction medium containing 0.1 mg/ml insulin (Sigma-Aldrich, USA), 1 µM dexamethasone, 1 mM 3-isobutyl-1-methylxanthine (IBMX) (Sigma-Aldrich, USA), and 0.2 mM indomethacin (Sigma-Aldrich, USA) for 28 days. Cells were stained with Oil Red O (Sigma-Aldrich, USA). Inverted phase contrast microscope was used to indicate the intracellular lipid droplets. Adipogenic mRNA markers were analyzed using RT-qPCR (n = 4).

### In vitro chondrogenic differentiation

Cells were cultured in 24-well plate (50,000 cells/well) with growth medium for 24 h and then maintained in chondrogenic induction medium supplemented with 0.1 µM dexamethasone (Sigma-Aldrich, USA), 50 mg/ml L-ascorbic-2-2phosphate (Sigma-Aldrich, USA), 40 mg/ml L-proline (Sigma-Aldrich, USA), 1% of insulin-transferrin-selenium (ITS) supplement (Thermo Fisher Scientific, USA), 15% FBS, 10 ng/ml of transforming growth factor (TGF)-β3 (Sigma-Aldrich, USA), and 2% Antibiotic–Antimycotic supplement for 21 days. Cells were stained with Alcian blue. Chondrogenic mRNA markers were analyzed using RT-qPCR (n = 4).

### In vitro osteogenic differentiation

The osteogenic differentiation protocol was performed according to previously published reports^80,81^. Briefly, cells were seeded onto 24-well culture plate (Corning, USA) in a concentration of 3.5 × 10^4^ cells/well and maintained in osteogenic induction medium for 14 days with routine 48-h substitution. Osteogenic medium was growth medium supplemented with 50 mg/mL ascorbic acid (Sigma-Aldrich, USA), 100 nM dexamethasone (Sigma-Aldrich, USA), and 10 mM β-glycerophosphate (Sigma-Aldrich, USA). Cells cultured in growth medium were utilized as the undifferentiated control.

### Alkaline phosphatase activity assay

The alkaline phosphatase activity was measured at 14 day after osteogenic induction according to previous reports^80,82^. Cells were lysed in lysis buffer. The lysed samples were incubated with *p*-nitrophenol at 37 °C for 15 min. The absorbances were read at a wavelength of 410 nm, then calculated for ALP activity using standard curve. Total protein concentrations were measured using Qubit according to manufacturer’s protocol (Thermo Fisher Scientific, USA). The enzymatic activity was expressed as U/mg protein.

### Mineralization assay

Deposited mineralization was examined by Von Kossa staining. The samples were gently washed with PBS and fixed in methanol for 10 min. Next, cells were washed with distilled water and incubated with 1% silver nitrate solution (Sigma-Aldrich, USA) under UV light for 30 min. After several washes using distilled water, unreacted silver was removed with 5% sodium thiosulfate (Sigma-Aldrich, USA) for 5 min. Images of the stained mineralizing nodules on the plate were obtained using an inverted microscope. Areas of mineralized nodule represented as strong brown-black compacted nodule were measured and normalized with total area using ImageJ program (NIH, USA). The results were presented as mineralized area ± standard deviation (SD)^83^ (n = 5).

### Protein extraction and in-solution digestion

Samples were lysed with lysis buffer containing protease inhibitor (Thermo Fisher Scientific, USA) and 5% sodium deoxycholate (SDC), then homogenized by sonicator. Protein concentrations were measured using bicinchoninic acid (BCA) protein assay (Thermo Fisher Scientific, USA). Protein samples (400 µg per sample) were mixed in 100 mM tetraethylammonium bromide (TEAB) (Thermo Fisher Scientific, USA) and incubated at 56 °C at 300 rpm for 1 h. Next, these samples were alkylated with iodoacetamide (IA) in a dark room for 30 min, mixed with 200 mM tris(2-carboxyethyl)phosphine (TCEP), and added cold methanol, and incubated overnight at − 20 °C. Samples were centrifuged at 8,000 rpm for 10 min and resuspended with 100 mM TEAB. The protein samples were incubated with trypsin at a ratio of 1:50 at 37 °C for 16 h. The quantity of tryptic peptides was measured with the Pierce Quantitative Fluorometric Peptide Assay (Thermo Fisher Scientific, USA). The peptide samples were collected at − 80 °C.

### In-solution dimethyl labeling and fractionation

The digested samples were reconstituted in 100 mM TEAB. The peptide samples of control group (cBM-MSCs and cDPSCs) and osteogenic induction groups (cBM-MSCs and cDPSCs) at day 7 and 14 were labeled with formaldehyde isotope including light reagents (formaldehyde and cyanoborohydride), medium reagents (formaldehyde-d2 and cyanoborohydride), and heavy reagents (deuterated and 13C-labeled formaldehyde and cyanoborodeuteride), respectively, at room temperature for an hour. Each isotope labeled sample was quenched by adding ammonia solution and formic acid. Three labeled-peptide samples were mixed. To reduce complexity, the complex mixture samples were separated into 10 fractions using the Pierce High pH Reversed-Phase Peptide Fractionation Kit (Thermo Fisher Scientific, USA). Elution samples of each fraction were evaporated the liquid content to dryness using vacuum centrifugation. Dry samples were resuspended in formic acid before LC–MS/MS analysis.

### LC–MS/MS and analysis

Before MS injection, the fractionated peptides were resuspended to a final volume of 15 µl in 0.1% formic acid (Sigma-Aldrich, USA). The samples were analyzed by an EASY nLC1000 system (Thermo Fisher Scientific, USA) connected to a Q-Exactive Orbitrap Plus mass spectrometer (Thermo Fisher Scientific, USA) supplied with a nano-electrospray ion source (Thermo Fisher Scientific, USA). Next, the peptide samples were eluted in 5–40% acetonitrile for 70 min and 40–95% acetonitrile for 20 min in 0.1% FA by using flow rate 300 nl/min. The full MS1 scan procedures employed a resolution at 70,000 and MS2 scan at 17,500. To select the target peak, range from 350 to 1400 m/z from MS scan was identified by using Proteome Discoverer™ Software 2.1 (Thermo Fisher Scientific, USA). The measures were set including digestion enzyme (trypsin), maximum miss cleavage (2) , maximum modification (4), fixed modification (carbamidomethylation of cysteine, + 57.02146 Da), dimethylation of N-termini and lysine (light, + 28.031300 Da, medium, + 32.056407 Da and heavy, + 36.075670 Da), and variable modifications (oxidation of Methionine, + 15.99491 Da). The relative MS signal intensities of dimethyl labeled peptides were analyzed by Proteome Discoverer™ Software. The mean and standard deviation of fold change from five replicates were calculated to Log2 value of the normalized ratio.

### Bioinformatics

The listed proteins were implemented to analyze by the online resource database for annotation, Reactome (https://reactome.org/) and DAVID (https://david.ncifcrf.gov/). These databases provided intuitive bioinformatics tools to categorize and interpret the proteins from the control group and osteogenic induction on day 7 and 14 by cBM-MSCs and cDPSCs during osteogenic differentiation.

### Hierarchical Clustering and level protein expressions

On day 7 and 14 post-induction, the protein expression levels were calculated as Log2 normalized ratio, by normalizing with undifferentiated control group (day 0). The relevant proteins found less than 3 from 5 replicates were excluded. Then, the mean and standard deviation of fold change across all 5 biological replicates were determined. The proteins were clustered and showed as the heatmap or cluster map with Row Dendrogram and Column Dendrogram. The levels of significant protein expressions were reported as fold-change number. Color scale was used for reflecting upregulation (red) and downregulation (blue) of protein expression after osteogenic induction at day 7 and 14, by normalizing the data with the undifferentiated control (day 0).

### Validation assay for potential signaling

To validate the relevance of potential signaling on osteogenic differentiation potential by cBM-MSCs and cDPSCs, specific inhibitors regarding each signaling pathway were employed including Wnt canonical inhibitor (Dkk-1, 100 ng/ml), Notch inhibitor (DAPT, 25 µM), TGF-beta receptor complex inhibitor (SB-431542, 4 µM), and BMP-2 signaling inhibitors (noggin, 0.2 µg/ml and dorsomorphin, 4 µM ). Analysis of matrix mineralization by *Von Kossa* staining was utilized at day 7 and 14 post-induction, and it was compared with osteogenic control.

### Statistical analyses

Four biological replicates were used for analyzing of ALP activity and osteogenic mRNA expression. The statistical analysis was performed using SPSS Statistics (IBM, USA). To compare two independent groups, the Mann Whitney U test was employed. Statistical difference was recognized when *p* value < 0.05.

Five biological replicates were used for analyzing of proteomics data. The mean and standard deviation of fold change from five replicates in each cell were presented as Log2 value of the normalized ratio. The significant proteins were called when they expressed at least 3 from 5 replicates. Significant difference between groups was determined by unpaired *t*-tests with *p* value < 0.05.

## Ethics statement

This study was approved by the Institutional Animal Care and Use Committee (IACUC), Faculty of Veterinary Science, Chulalongkorn University (Animal Use Protocol No. 1531038).

## Data Availability

The mass spectrometry proteomics data, including annotated spectra for all modified peptides and proteins identified on the basis of a single peptide, have been deposited to the ProteomeXchange Consortium via the PRoteomics IDEntifications (PRIDE) partner repository with the dataset identifier PXD015512 (Username: reviewer94686@ebi.ac.uk, Password: aai4ials).
